# Hydrogel Encapsulation of Mesenchymal Stem Cells and Their Derived Exosomes for Tissue Engineering

**DOI:** 10.3390/ijms22020684

**Published:** 2021-01-12

**Authors:** Parisa Khayambashi, Janaki Iyer, Sangeeth Pillai, Akshaya Upadhyay, Yuli Zhang, Simon D. Tran

**Affiliations:** McGill Craniofacial Tissue Engineering and Stem Cells Laboratory, Faculty of Dentistry, McGill University, 3640 University Street, Montreal, QC H3A 0C7, Canada; parisa.khayambashi@mail.mcgill.ca (P.K.); janaki.iyer@mail.mcgill.ca (J.I.); sangeeth.pillai@mail.mcgill.ca (S.P.); akshaya.upadhyay@mail.mcgill.ca (A.U.); yuli.zhang@mail.mcgill.ca (Y.Z.)

**Keywords:** mesenchymal stem cell, exosome, hydrogel, osteogenesis, angiogenesis, tissue engineering, biomaterial

## Abstract

Tissue engineering has been an inveterate area in the field of regenerative medicine for several decades. However, there remains limitations to engineer and regenerate tissues. Targeted therapies using cell-encapsulated hydrogels, such as mesenchymal stem cells (MSCs), are capable of reducing inflammation and increasing the regenerative potential in several tissues. In addition, the use of MSC-derived nano-scale secretions (i.e., exosomes) has been promising. Exosomes originate from the multivesicular division of cells and have high therapeutic potential, yet neither self-replicate nor cause auto-immune reactions to the host. To maintain their biological activity and allow a controlled release, these paracrine factors can be encapsulated in biomaterials. Among the different types of biomaterials in which exosome infusion is exploited, hydrogels have proven to be the most user-friendly, economical, and accessible material. In this paper, we highlight the importance of MSCs and MSC-derived exosomes in tissue engineering and the different biomaterial strategies used in fabricating exosome-based biomaterials, to facilitate hard and soft tissue engineering.

## 1. Tissue Regeneration and MSCs

Bone regeneration for critical size defects is challenging, and even the most commonly used approaches in bone reconstruction, such as autologous and allogenic bone grafts, do not meet all the requirements of a bioactive material [1]. For autologous grafts, the quantity of the harvested bone tissue is limited, and the procedure is sometimes associated with increased donor site morbidity [1]. Failures due to mechanical instability and immunological rejection after the surgery paved the way for the development of alternative techniques for bone regeneration and defect repair [1]. Tissue engineering deals with the combined application of principles of life sciences and engineering towards understanding the structural and functional relationship in physiological and pathological tissues, involving bioactive materials [2]. A biomaterial can be described as any natural or synthetic substance or combination of substances that, when applied intimately into a functional system, autonomously replaces and restores the tissues of the body [3].

This multidisciplinary science uses the properties of a porous biocompatible and biodegradable material in the form of two dimensional or three-dimensional scaffold or template upon which the cells are seeded to promote growth in the tissue microenvironment [2]. Biomaterials either act as an in vitro template to aid in tissue engineering, with adequate cell–cell interaction and growth factors, or as an aid in transplanting the regenerated tissue in vivo to integrate structurally and functionally with the system [2]. Biomaterials commonly encountered in tissue engineering comprise of natural and synthetic polymers, and ceramics [2]. Three-dimensional hydrophilic polymers, such as hydrogels, have emerged as a bioactive scaffold material in the recent past, which are commonly used in drug delivery and cell encapsulation [4].

Cell-encapsulated hydrogels demonstrate prolonged fundamental and operational consistency and are widely applied in regenerative therapy [4]. The developments in cell-encapsulated hydrogel therapies have been improved by the heightened ease of using mesenchymal stem cells (MSCs) with them [4]. When a combination of stem cells and hydrogels are used, certain considerations are necessary. For instance, when MSCs are used in three-dimensional (3D) microenvironments, their differentiation efficiency into osteocytes, hepatocytes, or adipocytes is increased as compared to two-dimensional (2D) cultures [4]. MSCs also have shown enhanced differentiation capacity when cultured in proximity to other cells, such as hematopoietic stem cells (HSCs) and human umbilical vein endothelial cells (HUVECs) [4]. The cell encapsulation method is also reportedly dependent on the shape and size of the vehicle that delivers the MSCs [4]. The 3D environment, whether it is a hydrogel or bioprinted microfluid droplets, is required to be tailored to the target tissue for optimal tissue regeneration [4].

In this regard, the MSCs’ secretions have gained special attention as a regenerative tool compared to other cell-based therapies [5]. Initially identified in the 1960s, MSCs were first described as spindle-shaped cells originating from bone marrow that could regulate the quiescence and self-renewal of hematopoietic stem cells through the release of paracrine factors [6]. These cells are rare, heterogenous, and, in addition to bone marrow, have been successfully isolated from placenta, amniotic fluid (AF), umbilical cord blood (CB), Wharton’s jelly (WJ), and adipose tissue (AD) [7]. MSCs are easy to isolate from adult tissues, have a large capacity for ex vivo expansion, and have proven to be efficiently therapeutic in many diseases [7].

The International Society for Cellular Therapy has released the three minimal requirements to define multipotent MSCs: first, MSCs being plastic-adherent; second, expressing CD105, CD73, and CD90 while not expressing CD45, CD34, CD14 or CD11b, CD79α or CD19, and HLA-DR surface molecules; and third, having differentiation capacity into osteoblasts, adipocytes, and chondroblasts in vitro [7,8,9].

Treating sites of injury using MSCs has its own disadvantages. An estimated more than 99% of these cells get trapped in the spleen, lungs, and liver [10,11], and those that do approach the targeted tissue have a low survival time and are predisposed to cause thrombosis, fever, and tumors [12]. An alternative approach would be to consider the paracrine mechanisms of MSCs, especially the nanometer vesicles, exosomes, as the source of biomaterials for tissue repair (Figures 3A and 4) [12]. Recent studies have identified MSC exosomes as the mediator in carrying the restorative agents [10]. In addition to lowering the potential risks of cell-based therapy, processing, and storage conditions, exosomes are less sensitive than MSCs [13]. Exosomes will not self-replicate and consequently would not cause tumor formation [13].

## 2. MSCs and Exosomes

Although the benefits of exosomes are well-known, the drawbacks of delivering a therapeutic dosage of exosomes, especially through systemic injections, may outweigh their advantages [14]. As for the biological effects of exosomes to be prompted, they are required to be internalized via endocytosis by the targeted cell, otherwise they rapidly would be cleared from the blood circulation and may even accumulate in the liver, spleen, lungs, and gastrointestinal tract [14]. Direct intravenous, intraperitoneal, or subcutaneous injections of exosomes can mount a reaction by macrophages in the reticuloendothelial system, leading to their rejection. Bodily and topical applications on skin or ocular surfaces has shown short half-lives after interacting with sweat, tears, and the epithelial barrier (tight junctions) [14]. On the other hand, the difficulties in exosome purification and mass-scale production emanate from the expensive manufacturing protocols that require consistency and purity of nanometer-sized biomaterials [14]. Therefore, delivering exosomes entails a more efficient method to elude from being cleared by the host body.

### 2.1. Characterization of Exosomes

**Figure 1:**
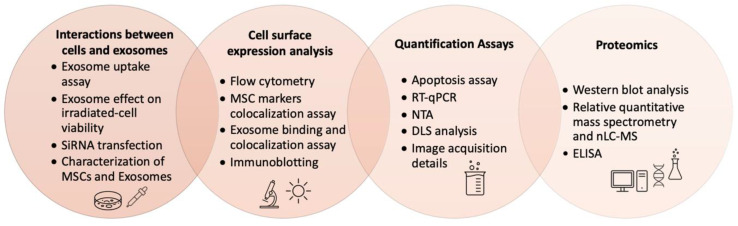
A summary of commonly used methods to characterize exosomes: exosome uptake assay [15], exosome effect on irradiated-cell viability [15], SiRNA transfection [15], immunoblotting [15], cell surface expression analysis (flow cytometry) [15], MSC markers colocalization assay [15], exosome binding and colocalization assay [15,16,17], image acquisition details [18,19,20], apoptosis assay [19], quantitative reverse transcriptase polymerase chain reaction assay (RT-qPCR) [21], nanoparticle tracking analysis (NTA) [21,22,23], dynamic light scattering (DLS) analysis [16], Western blot analysis [24], relative quantitative mass spectrometry and nano-liquid chromatography mass spectrometry (nLC-MS) [21], and ELISA [25].

As demonstrated in Figure 1 and Figure 3B, a plethora of methods are being tested to isolate and characterize exosomes from different types of cells (Figure 3A). Due to the size distribution (50–120 nm) and the delicate membranous nature of the exosomes, characterizing them before their involvement in biomaterials is critical to optimize the desired effect in the target tissue [15]. These characterizations consist of assays to evaluate the interactions of the exosomes with the surrounding tissue, surface markers, their proteomics profile, their morphology, and size [15]. From 1 mL of culture medium, typically less than 1 µg of exosome proteins could be isolated whereas the suggested therapeutic dosage for humans would require 100–1000 times this value [14]. Thus, the need for a biocompatible, bioactive, and biodegradable material for delivering therapeutics with exosomes has brought the attention of biomedical science to porous hydrogels [14].

### 2.2. Exosomes and Biomaterials

Due to the reasons indicated earlier, the most pertinent application of exosomes in regenerative medicine is by conjoining them with a biomaterial [16]. Several studies have evaluated this combination; for instance, Shi et al. reported accelerated angiogenesis, neurogenesis, reepithelization, and collagen formation on investigating a chitosan/silk hydrogel sponge as a carrier for gingival MSC-derived exosomes [16]. In another study, human placenta-derived MSC exosomes, when encapsulated in a chitosan hydrogel, have also shown enhanced angiogenesis and tissue regeneration in a mouse hindlimb [26].

**Figure 2:**
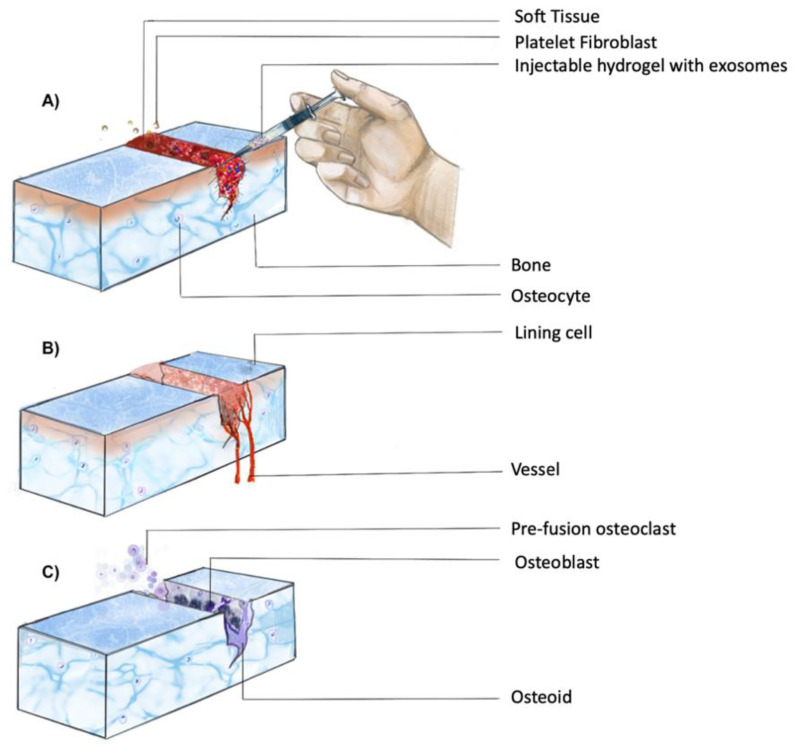
Temporal progression of fracture healing. Healing of a fracture involves a complex series of processes, which can be broadly divided into three phases: (**A**) inflammatory phase; (**B**) soft callus formation; (**C**) mineralization of callus and bone remodeling (adapted from Upadhyay et al. [29]).

As illustrated in Figure 2, in tissue regeneration, and more specifically during bone formation, osteoblastic cells begin to proliferate and produce an osteogenic matrix, leading to the formation of a new bone structure and an increased metabolic demand, which is reciprocated by an increase in the blood flow rate (BFR) and vascular density (VD) [27]. At this point, endogenous stem cells or its secreted exosomes have to be recruited to enhance neovascularization [28]. This recruitment is sensitive and essential, and its efficiency determines the success rate of procedures such as allograft tissues in bone reconstruction surgeries [27,28].

**Figure 3:**
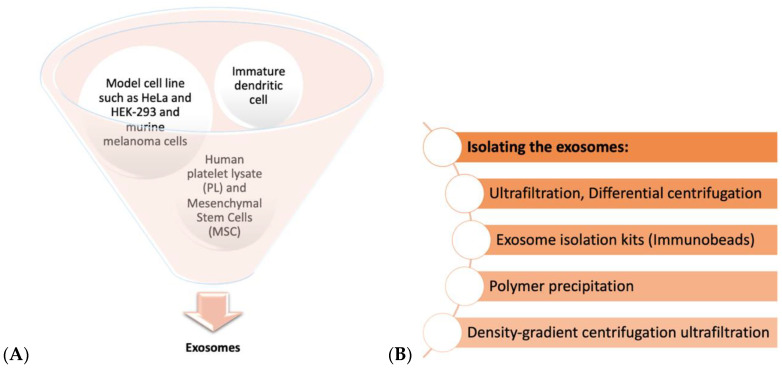
Summary of cell sources and methods used to isolate exosomes. (**A**) Sources of donor cells where exosomes have been isolated. (**B**) Methods of exosome isolation: ultrafiltration exosome isolation kits, polymer precipitation, differential centrifugation, and density-gradient centrifugation ultrafiltration [6,15,17].

**Figure 4:**
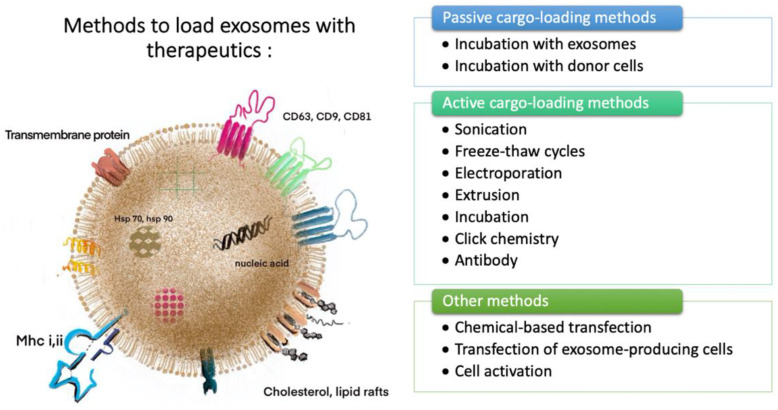
General scheme of a mesenchymal stem cell exosome and methods to load it with therapeutics. Left panel: Layout of an MSC-derived exosome containing cargos such as nucleic acids and proteins. The respective molecules and markers are shown on the surface of the exosome. Right panel: Methods to load the exosomes with therapeutics. Figure adapted from Hofmann et al. [37].

Perfecting the efficiency of a biomaterial to facilitate osteogenesis and angiogenesis is the driving force behind several studies incorporating biomaterials and tissue engineering. Therefore, finding the right cell type to isolate exosomes from (Figure 3A,B) and the corresponding method to characterize these exosomes are as important as discovering the suitable method to load them with therapeutics and embed them in the proper hydrogel (Figure 4). As depicted in Figure 3, exosome donor cells vary from the different types of cells, such as immature dendritic cells [21,30,31,32]; model cell lines, such as HeLa and HEK-293 and murine melanoma cells [30]; human platelet lysate (PL) [33]; and MSCs [34,35], see also [7,17,36].

As shown in Figure 4, methods to load the exosomes with therapeutics include passive [9] or active methods. Passive methods include incubation with exosomes [30,38] and incubation with donor cells [38]. Active methods include sonication [4,39], freeze–thaw cycles [38,39], electroporation [9,19,30], extrusion [38,39], incubation [38,39], click chemistry [38,39], and antibodies [38,39]. Methods such as chemical-based transfection [30], transfection of exosome-producing cells [30], and cell activation [30] are among the other techniques for loading exosomes with therapeutics.

## 3. Exosomes and Hydrogels

Hydrogels are three-dimensional polymers that are physically or chemically cross-linked in structure, function as biocompatible scaffolds, and demonstrate a strong affinity for water [4,40]. Hydrogels possess unique properties that can be exploited towards versatile biomedical applications, such as in [40,41]:

1. A hydrophilic porous structure—an ability to absorb and retain water while maintaining structural integrity, allowing the free diffusion of particulate materials.
2. Shear and compressive stress—Young’s modulus and atomic force microscopy of hydrogels determine the cellular migration and proliferation in biomaterial scaffolds.
3. Volume phase transition or gel-sol transition—depending on the nature and magnitude of the external stimuli, such as physical (electromagnetic fields, temperature, and pressure) and chemical (pH and ions), the hydrogels exhibit reversible volume changes.
4. Degree of flexibility—the structural lattice of the hydrogels formed by the crosslinked monomer–polymer networking, with covalent and non-covalent bonds, enhance their adaptability to the microenvironment, mimicking the tissue.
5. Degradability—the synchronized degradation of the hydrogels to support cellular growth in the tissue microenvironment.

Owing to this versatility, hydrogels mimic natural tissue, and hence have been widely applied and constantly modified to enhance different biomedical applications. Augmentation of the hydrogel scaffolds, to improve cellular migration, proliferation, and differentiation, promotes their application in tissue engineering, regenerative medicine, adhesive medicine, cell-encapsulation matrices, and drug-delivery systems [40,42,43].

Hydrogels have been widely used as a carrier for the sustained local drug delivery of treated exosomes, such as a chitosan/silk hydrogel sponge as a substrate for human gingival MSC-derived exosomes and human placental-derived MSCs [4,16,44]. The hydrophilic and cross-linking behavior of hydrogels facilitate their ability for controlled drug release, and have proven impactful in the fields of angiogenesis, osteogenesis, oncology, immunology, and pain management [40].

### 3.1. Hydrogel-Exosome Encapsulation Strategies

Development of smart biomaterials has unfastened the scope for drug encapsulation. This has led to efficient and sustained delivery of biomolecules in a site-specific manner, such as hydrogels. Using hydrogels has facilitated the process of harnessing the therapeutic benefits of exosomes in bone tissue engineering. There are three common strategies for encapsulating exosomes into a hydrogel matrix [14].

**Figure 5:**
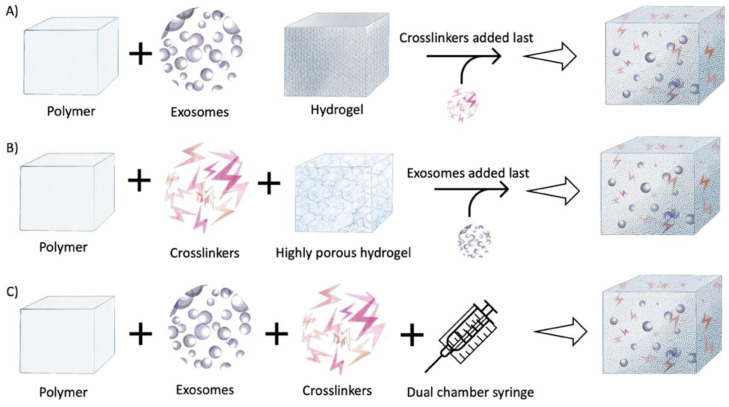
Approaches to encapsulate exosomes in hydrogels: (**A**) Combining the exosomes with polymers followed by addition of crosslinkers to induce gelation. (**B**) Physical incorporation of the hydrogels, or the “breathing” technique. (**C**) Mixing of the exosomes with both the polymers in solution and crosslinkers simultaneously. Adapted from Riau et al. 2019 [14].

1. Combining exosomes with polymers followed by addition of crosslinkers to induce gelation (Figure 5A). This technique was explained by Qin et al., where they used thiolated hyaluronic acid (HA), gelatin, and heparin as the main components. Bone marrow stem cell-derived exosomes were incorporated into this polymer and polyethylene glycol diacrylate (PEGDA) was used as a gelation agent [45,46]. This is based on covalent crosslinking of the active precursors. They are an attractive choice of exosome and cell encapsulation since they provide high tunability of the hydrogels, allowing control over the mechanical properties and degradation rate [47]. However, one common concern arises with the addition of new compounds, such as the crosslinkers, which can be potentially be cytotoxic to the biomolecules. One additional advantage with this technique is the use of the macromolecular monomers usually derived from the biocompatible polymers [48].

2. Physical incorporation of hydrogels or “breathing” technique (Figure 5B). This method involves two basic steps. First, the already swollen hydrogel is placed into a solvent that removes the water present in the hydrogel. The hydrogel is then soaked in an aqueous solution containing the exosomes, causing the breathing-in of the exosomes into the porous hydrogel [49]. This technique is based on the simple principle of smart hydrogels forming swollen structures when kept in water, but on exposure to solvents with lower polarity becomes collapsed and undergo a phase transition [50]. However, to use this technique, the pore size of the hydrogel should be moldable and larger than the exosomes or the stem cells that need to be encapsulated. Once inside, the loosely attached exosomes will leach out when exposed to the site of action [49].

3. Mixing of the exosomes with both the polymers in solution and crosslinkers simultaneously (Figure 5C). This leads to an in situ gelation, allowing targeted delivery of the exosomes, as described in the study by Wang et al., where they used adipose-derived exosomes with polypeptides for wound healing and skin regeneration [51]. This is usually achieved by a dual chamber syringe that can inject the hydrogel components with exosomes directly to the site. [14,46,48]. In situ gelation can be achieved by several mechanisms, such as UV irradiation, ion-exchange, pH change, and temperature changes [52]. This technique is highly notable in filling critical size defects with complex geometries, allowing good viability of the incorporated biomolecules. These injectable scaffolds will have the desired native tissue properties, and thus can function without external inducers [53].

### 3.2. Hydrogel Combinations for Exosome and Stem Cell Encapsulation

Sustained delivery of exosomes has been a widely studied research area in tissue engineering. Although in its infancy, the potential of hydrogel-based delivery systems is tremendous. Table 1 summarizes some of the most significant hydrogel-based biomaterial strategies used to incorporate exosomes and their parent stem cells in hydrogels for various applications in biomedicine.

To date, HA, gelatin, chitosan, and polypeptide-based hydrogels have been used for encapsulating exosomes from different cell sources [5,45]. Although the mechanism of embedding exosomes within hydrogels includes one of the three strategies described in Figure 5, minor modifications made to the principal technique improves the fabrication ease and allows efficient delivery of the exosomes. For example, Liu et al. used a photoinduced imine crosslinker by reacting the aldehyde groups, generated by the light irradiation of the O-nitro benzyl alcohol groups, to modified HA and amino acids on gelatin [5]. PGLA has been largely used as a material of choice for exosome encapsulation and a widely used biocompatible scaffold for tissue regeneration.

The mussel-inspired technique was used by Lee et al. for immobilization using pDA (polydopamine) to provide a more efficient coating on the PLGA substrate [63]. They immobilized the bone-forming peptides-1 (BFP-1) using this technique, which allowed a slow release of BFP-1 and showed that combining hASCs and PLGA/pDA can enhance bone formation [64]. Most recently, Wang et al. described a highly efficient injectable self-healing hydrogel fabrication using a Schiff base linkage for applications in severe wound healing [61]. They constructed a methyl-cellulose (MC)–chitosan (CS) combination of a hydrogel with placental cell-derived exosomes. The MC- and CS-grafted polyethylene glycol were synthesized using DCC (dicyclohexyl- carbodiimide) and EDC (1-(3-dimethylaminopropyl)-3-ethylcarbodiimide) reactions. Both these polymer solutions were mixed together along with exosomes to fabricate the complex hydrogel [61].

Scaffold production, using physical freeze drying and crosslinking methods, have provided good results in terms of cell encapsulation. However, Chen et al. described fabrication of a 3D-printed decellularized extracellular matrix (ECM) with gelatin methacrylate loaded with exosomes from MSCs using desktop-stereolithography technology [65]. This technique allowed the synthesis of radically oriented channels, which provides superior cartilage regeneration by directing the migration of the chondrocytes and thus repairing osteochondral defects [65].

Currently, all materials commonly used for exosome encapsulation are naturally derived owing to their ease of handling and fabrication, higher biocompatibility, and ability to simulate ECM-like conditions [65]. Although there might be harmful effects due to the presence of crosslinkers and residual peptides during hydrogel formation, they are highly formable and can be constructed for patient-specific needs, making them the key biomaterial for drug delivery and other tissue-regeneration applications [65]. Still, the challenges in their use include a lack of slow-releasing potential, inability to induce surface modifications, and timing the delivery of exosomes or bioactive molecules to coincide with the natural healing and regenerative processes [65].

### 3.3. Hydrogels and Exosomes in Hard Tissue Regeneration

Inducing hard tissue regeneration with a controlled release of drugs requires the presence of suitable cells as the foundation [66]. Bone regeneration is based on three key factors: stem cells, scaffolds, and growth factors [67]. Figure 2 depicts the cascade of events for healing of a fracture and consists of a complex physical process for delivering drugs and biomaterials [66]. Amongst the hard tissues, regeneration of cartilage remains challenging due to its avascular nature [68]. Given the high incidence of age-related diseases, such as osteoarthritis and injuries, it becomes imperative to look for alternative regenerative procedures [68].

Scaffolds and grafts have been used for ages for bone regeneration. Especially for hard tissue repair, artificial grafts and scaffolds offer the advantage of having less morbidity as there is no need for secondary surgery or donors. They are also cheaper and customizable to the required needs. There have been numerous advances to inculcate their inductive nature by fabrication of hybrid biomaterials with multipotential cells and their released factors. As depicted in Table 2, each biomaterial releases its secretory content at a different rate, and hence has a different potential clinical use.

Exosome-integrated scaffolds helped in restoring the mitochondrial function of degrading cartilage in an osteoarthritic cell model. Chen et al. identified the proteins associated with degrading mitochondrial function through protein enrichment analysis, which were less expressed in treated cells, leading to the rescue from osteoarthritic degradation [65]. Additionally, through transwell migration assays, it was found that the MA matrix with exosomes and ECM proteins had the maximum influence on chondrocyte migration [65].

Table 2 lists the studies that had tested additional parent cell types and combinations of assisted matrices with hydrogel compositions. One of the most commonly used compositions is PLGA, which is applied as a biocompatible scaffold for tissue regeneration. The challenge with PLGA is its lack of ability for slow-releasing surface modification. Such a feature becomes essential while timing the delivery of the exosomes or bioactive molecules to coincide with the natural healing and regenerative process. To overcome this issue, the release profile of the PLGA polymeric scaffolds was assessed by combining it with polydopamine (pDA), a mussel-inspired biomaterial that allows higher adhesions [60]. The exosome burst release from PLGA/pDA was recorded to be significantly slower (8 days) than the PLGA samples (4 days) [60]. In another study, Yang et al. found better release kinetics with 71.2% of exosomes being released over 14 days using hydroxyapatite (HAP) and hyaluronic acid–alginate gel (HA–ALG) hydrogels [59].

Each study entails a specific physiological pathway exploited for osteogenic induction. For instance, Li et al. used hASC-derived exosomes two days after osteogenic induction to potentiate the osteogenesis-promoting factor production [60]. In the same study, the chemotactic and proliferative effect on BMSCs were tested and it was found that during the phenotypic translation of the MSCs, its chemotactic and proliferative effects may be lost [60]. Another important pathway found to be involved with exosome release from stem cells is the PI3Akt pathway, as the exosomes derived from human-induced pluripotent stem cells (hIPSCs), integrated into tricalcium phosphate, were seen to enhance bone regeneration through the phosphoinositide 3-kinase (PI3Akt) pathway [1,71].

### 3.4. Hydrogels and Exosomes in Soft Tissue Regeneration

Soft tissue regeneration is required in cases of delayed wound healing as in diabetic patients or large soft tissue wounds like burns [72]. Biomaterials for soft tissue wound healing are required to be able to adapt to the uneven morphology and mobility of the defect and also to have adhesiveness to the surrounding tissue to offer complete coverage as well as prevent exposure of the healing site to the environment (Table 3) [72]. The characteristics of assisted matrices for soft tissue regeneration include having a thermally responsive gelation, water retaining ability, as well as antibacterial and adhesive properties [51,73]. Hydrogels, such as FHE, offer these properties and, when loaded with exosomes, have shown enhanced angiogenic potential [51,73]. Regenerative medicine has offered countless approaches for assisted wound healing [72]. With the recently identified advantages of exosomes, efforts are being made to integrate past and newer biomaterial scaffolds with these multiple factor-packed small molecules [72]. Biomaterials for soft tissue wound healing are required to be able to adapt to the uneven morphology and mobility of the defect and also to have adhesiveness to the surrounding tissue to offer complete coverage as well as prevent exposure of the healing site to the environment. This adaptability ensures the most efficient biomimetics and can be offered by bio-responsive smart materials.

As demonstrated in Table 3, Wang et al. studied the effect of both free exosomes and those encapsulated in an FHE hydrogel on HUVEC cells and found that sustained release from the hydrogel supported a better growth by being less toxic to the cells and providing the required stimulus [51,73]. The FHE (F127/OH-EPL) hydrogel offered a tissue-responsive behavior by having thermally responsive gelation, water retaining ability, as well as antibacterial and adhesive properties [51,73]. Loading with exosomes has shown an enhanced angiogenetic potential in a diabetic mice model [51,73]. They successfully demonstrated good injectability, self-healing, antibacterial activity, and stimuli-responsive exosome release [51,73]. Similarly, a self-healing hydrogel consisting of a methyl-cellulose–chitosan hydrogel with placental mesenchymal stem cell (PMSC)-derived exosomes was also shown to be capable of diabetic wound healing, where neo tissue formation with similarity to natural skin was achieved [61].

Skin regeneration has long been associated with aesthetic and plastic surgery [76]. Duncan et al. recently added exosomes with polydioxanone (PDO) threads in micro lifting surgery, which enhanced the bio stimulatory potential, and thus resulted in a faster clinical outcome [77]. Neural regeneration is also an exciting area where exosomes have been tested and proven helpful [78,79]. Exosomes from hGMSCs with chitin conduits were experimented on in a rat model by Rao et al., and the results showed improved repair of sciatic nerve damage [75].

## 4. Alternative Methods to Exosome Delivery

Aside from using exosome-embedded hydrogels to achieve a topical and sustained drug release effect, when exosomes are not encapsulated in hydrogels, they are delivered intravenously or topically [80]. Sun and co-authors [80] have designed a delivery method that can topically release exosome at the target sites. They also intravenously injected exosomes together with a SonoVue^TM^ microbubble (Bracco Imaging) into mice, and then targeted the destruction of these microbubbles by ultrasound [80]. They proved that the ultrasound-targeted microbubble destruction (UTMD) significantly increased the exosome infiltration and endocytosis. This method can be used as an alternate strategy for exosome delivery and endocytosis enhancement, but the disadvantage is that the exosomes would be quickly metabolized by the blood circulation system, causing a low utilization rate of the exosomes [80].

Multifunctional mesoporous bioactive glasses are also used for delivering therapeutic ions to the site of regeneration [81]. Mesoporous silica nanoparticles (MSNs) as a drug-delivery system have recently been used for bone tissue engineering [82]. These nanoparticles have a high specific surface area and pore volume, which allows for high loading of a drug and the controlled release of the drug from days to even weeks [82]. Among other nanoparticles, using icariin loaded on micro/nano hybrid structured hydroxyapatite granules, has shown the potential for repairing femoral defects [83]. In segmental bone repair, locally applied granulocyte colony-stimulating factor (G-CSF) has shown to enhance bone regeneration via neovascularization and osteogenesis [84]. In a similar context, using a graphene-based miRNA transfection drug-delivery system, Dou and colleague discovered that the platelet-derived growth factor secreted by pre-osteoclasts (POC) can enhance bone mineral density, bone volume, and bone vascularization [85].

## 5. Conclusions

Tissue engineering with its requirements has created several new regenerative tools and biomaterials. Among the plethora of biomaterials, those combining hydrogels and stem cell therapy have shown promising results. Due to the numerous advantages of MSCs and their secretions (i.e., exosomes), several hydrogel formulations, such as PLGA, pDA, alginate, chitin PLGA/pDA, and FHE, have been reported to induce hard and soft tissue regeneration.

## Figures and Tables

**Table 1:** Summary of the most commonly used hydrogel-based systems incorporating exosomes/stem cells, with their corresponding applications.

Material Used	Type of Cells/Exosomes	Application	Reference
Adamantane and β-cyclodextrin-modified hyaluronic acid (HA) hydrogel	Bone marrow-derived endothelial progenitor cells	Cardiac regeneration	[54]
Alginate hydrogel	Blood plasma	Skin regeneration	[55]
Chitosan based hydrogel with ultrasound treated silk fibroin	Human umbilical cord MSCs derived exosomes containing miRNA-675	Cardiac/Muscle regeneration	[56]
Chitosan biopolymer (hydrogel-exosome composite)/with hydroxyapatite nanoparticles	Synovium-derived MSCs with miRNA-126-3p and exosomes	Wound healing	[57,58]
Chitosan/Silk hydrogel sponge	MSC-derived exosomes	Wound healing	[16]
Hydroxyapatite-embedded hyaluronic acid-Alginate hydrogel	Human umbilical cord mesenchymal stem cells-derived exosomes	Bone regeneration	[59]
Injectable Chitosan hydrogel	MSC-derived exosomes	Ischemia	[26]
Photoinduced imine crosslinking hydrogel glue-based acellular tissue patch	SC-ex (stem cell exosomes)	Cartilage regeneration	[5]
Polydopamine-coating polylactic-co-glycolic acid (PLGA) (pDA) scaffolds	Adipose-derived stem cells (hASCs)	Bone regeneration	[60]
Polypeptide based FHE hydrogels (Pluronic F127/OHA/EPL)	Adipose-derived MSCs exosomes	Wound healing and skin regeneration	[51]
Self-healing methylcellulose chitosan hydrogel	Placental MSC-derived exosomes	Wound healing	[61]
Thermosensitive chitosan hydrogel	hP (human placenta)-derived MSCs	Muscle regeneration	[62]

**Table 2:** Stems cells combined with hydrogel matrices and their corresponding clinical/in vivo uses in hard tissue regeneration.

Parent Cell Type	Assisted Matrix	Potential Clinical Use/In Vivo Experiments	Release Kinetics	References
hASC	PLGA/pDA	Calvarial defects in mice	87% in 8 days	[60]
hBMSC	PCL/GSNO	Barrier membrane for tissue regeneration	Not mentioned	[69]
hBMSC	GelMA	Cartilage repair in osteoarthritis	56% left after 14 days	[65]
hGMSCs	PLA	Rat calvarial defects	Not mentioned	[70]
hIPSC	PIC hydrogel glue	Rabbit articular defect	90% left in gel after 14 days	[5]
hIPSC	Β-TCP	Calvarial defects in mice	Burst release	[71]
hUCMSC	Injectable HAP—embedded in an in situ crosslinked HA–ALG hydrogel system	Calvarial defects in mice	71.2% in 14 days	[59]

hUCMSC (human umbilical cord mesenchymal stem cells); HA–ALG (hyaluronic acid–alginate); HAP (hydroxyapatite); hASC (human adipose derived stem cells); PLGA (poly (lactic-co-glycolic acid)) with a polydopamine-coating (pDA); hIPSC (human-induced pluripotent stem cells); PIC (photo-induced imine crosslinking gel); β-TCP (tri-calcium phosphate); hBMSC (human bone marrow stem cell); PCL (polycapronolactone) with GSNO (S-nitrosoglutathione); GelMA (methacrylate); hGMSCs (human gingival mesenchymal stem cell; PLA (polylactide).

**Table 3:** Stems cells combined with hydrogel matrices with their corresponding clinical/in vivo uses in soft tissue regeneration.

Parent Cell Type	Assisted Matrix	Potential Clinical Use	Reference
hASC	FHE hydrogel	Severe diabetic wound healing	[51]
hASC	Alginate	Skin grafts	[74]
hGMSCs	Chitin	Nerve repair	[75]
hGMSCs	Chitin	Diabetic wound healing	[16]
PMSC	MC/Chitosan	Diabetic wound healing	[61]

FHE (Puronic F127), oxidative hyaluronic acid (OHA), and poly-l-lysine (EPL); PMSC (placental mesenchymal stem cell derived).

## Abbreviations

AD	adipose tissue
AF	amniotic fluid
BFP-1	bone-forming peptides-1
BFR	blood flow rate
BMPs	bone morphogenetic proteins
CB	cord blood
CS	chitosan
CSDs	critical size defects
DCC	dicyclohexyl-carbodiimide
DLS	dynamic light scattering
ECM	extracellular matrix
EDC	1-(3-dimethylaminopropyl)-3-ethylcarbodiimide
FGF	fibroblast growth factor
G-CSF	granulocyte colony-stimulating factor
Gel MA	methacrylate
GSNO	S-nitrosoglutathione
HA	hyaluronic acid
HA–ALG	hyaluronic acid–alginate
HAP	hydroxyapatite
hASC	human adipose-derived stem cells
hBMSC	human bone marrow stem cell
hGMSCs	human gingival mesenchymal stem cell
hIPSC	human-induced pluripotent stem cells
HMG-CoA	3-hydroxy-3-methyl-glutaryl coenzyme A
hUCMSC	human umbilical cord mesenchymal stem cells
IGF	insulin-like growth factor
MBG	mesoporous bioactive glasses
MC	methylcellulose
MSCs	mesenchymal stem cells
MSNs	mesoporous silica nanoparticles
NTA	nanoparticle tracking analysis
OHA	oxidative hyaluronic acid
PCL	polycapronolactone
pDA	polydopamine
pDA	polydopamine-coating
PDGF	platelet-derived growth factors
PECL	poly (epsilon-caprolactone)
PEGDA	polyethylene glycol diacrylate
PIC	photoinduced imine cross-linking gel
PLA	polylactide
PLGA	poly (lactic-co-glycolic acid)
PMSC	placental mesenchymal stem cell derived
POC	preosteoclast
S1P	sphingosine 1-phosphate
TCP	tri-calcium phosphate
TGF	transforming growth factor
VD	vascular density
WJ	Wharton’s jelly
